# The Physiological Effects of Alcohol and Tobacco upon the Human System

**Published:** 1857-04

**Authors:** William A. Hammond

**Affiliations:** Assistant Surgeon, U. S. Army.


### ARTICLE IX.

*The Physiological Effects of Alcohol and Tobacco upon the Human System.* By WILLIAM A. HAMMOND, M. D., Assistant Surgeon, U. S. Army.

THE present paper is intended to exhibit the action of alcohol and tobacco upon the system generally, and, more especially, upon the important functions concerned in the metamorphosis of tissue.

The experiments illustrative of the effects of these substances were performed upon myself, and were conducted with all the care and accuracy which my limited facilities permitted. Those only who are familiar with investigations of this character can appreciate the time and labor necessary to conduct them properly, and but for the improved and extended system of volumetric analysis now so much employed in physiological chemistry, I should probably have been compelled to refrain from inquiries necessarily tedious at the best, but incomparably more so when the older methods of quantitative analysis are observed. Yet, when we reflect, however tiresome and even disgusting physiological investigations often are, that it is only by actual experiments we can ever hope to lay the foundations of true physiological science, we can well afford, for the sake of accomplishing so noble an end, to labor cheerfully on, even though the way be not so nice as we might desire. The day of extravagant theories, unsupported by observation, has gone by, and he who has nothing better to offer than the unsustained creation of a dreamy mind, meets with but little attention, and merits still less than he receives.

The influence of *alcohol* upon the human system has recently been the subject of thorough investigation by Dr. Böcker, who, with a degree of zeal worthy the importance of the inquiry, performed a series of experiments upon himself which have rarely been excelled for completeness and accuracy; but as the conclusions derived from his observations have met with the opposition of several distinguished physiologists, additional investigation seemed not altogether uncalled for.

The experiments relating to the action of *tobacco* detailed in the present paper, are believed to be the first of the character which have been performed. Physicians have heretofore been content to decry its use as uniformly injurious, without seeking for a reason for its deleterious influence, or even attempting to show that it was so generally pernicious as they believed. That both it and alcohol, when used with discretion, are capable of exercising highly beneficial effects upon the organism, will be abundantly shown from the ensuing experiments. Their influence, however, is not constantly advantageous, and when employed under circumstances which do not justify their use (like many other articles of food of much less doubtful reputation,) they may produce results which are far from conducive to health.

My own system was, I conceived, well calculated to exhibit the action of these agents satisfactorily. Not being in the habit of using either of them, I was peculiarly sensitive to their influence, and was able to perceive effects which, in a person more habituated to their use, might have escaped observation.

My manner of living during the succeeding investigations was as follows:

I arose every morning at six o'clock, and retired to bed every night at eleven. I was thus awake seventeen hours, and asleep seven. The seventeen waking hours were thus appropriated: ten were assigned to study of as uniform a character as possible, five to daily duties, recreation, &c., and two to a uniform system of physical exercise. This course was rigorously insisted on throughout the whole of the experiments with both alcohol and tobacco.

*Alcohol.*—I had three objects in view in investigating the action of this agent.

1. To observe its effects upon a system in which the weight of the body was *maintained at a nearly uniform standard* by a sufficiency of food.

2. To ascertain its influence upon an organism where the body *lost weight* from a deficiency of food.

3. To determine its action upon a system where the body *gained weight* from an excess of food.

The experiments under these heads related to the weight of the body, the quantity of carbonic acid and aqueous vapor expired in respiration, the weight of the fæces, the quantity of the urine, and the amount of its free acid, urea, uric acid, chlorine, and phosphoric and sulphuric acids. Besides these special determinations, I observed minutely every circumstance connected with my general health which could reasonably be ascribed to the action of the alcohol. I regret that I had no means at my command for accurately determining the amount of the cutaneous transpiration. Wherever this was sensibly affected it is noticed, but the liability to error when judging solely from sensation must not be forgotten.

The weight of the body was taken every day at 7 A. M., and at 2 and 10 P. M. The means of these observations are given in the tables. The carbonic acid and aqueous vapor exhaled from the lungs were determined by causing the expired air to pass through a tube containing chloride of calcium, and then through a saturated solution of baryta contained in two Woulfe's bottles. The excess of weight of the chloride of calcium tube indicated the amount of aqueous vapor, and from the quantity of carbonate of baryta formed, the carbonic acid was estimated. These determinations were made at 9 A. M., and at 2 and 10 P. M., and were continued one minute. From the mean of these observations the quantity for the day was calculated. As Vierordt has shown that the rate of respiration exercises a material effect upon the quantity of carbonic acid expired, I breathed during these observations uniformly fourteen times per minute, which is about the average natural frequency of my respiration. As comparative results were what I most desired, this method of estimation was sufficiently accurate.

The fæces was weighed at 8½ A. M., immediately after their evacuation. The whole quantity of urine passed during the twenty-four hours was accurately measured. The acidity of this fluid was determined by a test solution of ammonia, and was estimated as oxalic acid, and the urea, uric acid, chlorine, and phosphoric and sulphuric acids were ascertained as in the experiments recorded in the April number of this journal.

In the following tables the weight of the body is given in pounds and decimals, the fæces in ounces and decimals, and the quantity of urine in fluid-ounces and decimals. The weights of all the other substances are stated in grains and decimals. This system, though not so convenient as the French, has the advantage of being more commonly understood in this country, where the latter method is not yet generally adopted.

In the series of investigations previously detailed, ten days was the period fixed upon for obtaining average results. Further experience has, however, convinced me, that these can be obtained of sufficient accuracy in five days, and, where so many observations have to be made, the saving of time is an item not to be disregarded.

1. The action of *alcohol* where a uniform weight of the body was preserved.

After several trials, I found that food of the quality and quantity stated below, and taken as specified, kept up my weight to a nearly perfectly fixed standard.

I breakfasted at seven, lunched at one, and dined at five. At breakfast I ate five ounces of beefsteak, eight of bread, one half ounce of butter, and ten grains of salt, and drank six ounces of strong coffee, containing two drachms of cream, and two of white sugar. At luncheon, I ate three ounces of cold roast beef, six of bread, two drachms of butter, and twenty grains of salt. At dinner, I took six ounces of strong beef soup, eight of roast beef, four of boiled beets, four of bread, two drachms of butter, half a drachm of salt, and drank four ounces of coffee. In addition to this food, I drank daily forty-eight ounces of water, twelve at each meal, and twelve immediately before going to bed.

I thus took daily into my system sixteen ounces of beef, eighteen of bread, six of soup, four of beets, one of butter, one drachm of salt, two of cream, and two of sugar, and drank ten ounces of coffee and forty-eight of water.

The following table contains the results of the experiments instituted under the foregoing conditions. The temperature of the atmosphere during their continuance was in the mean 73.06° Fahrenheit.

| | Weight of body. | Carbonic acid. expired. | Aqueous vapor expired. | Fæces. | URINE. | | | | | | |
|---|---|---|---|---|---|---|---|---|---|---|---|
| | | | | | Quantity. | Free acid. | Urea. | Uric acid. | Chlorine. | Phosphoric acid. | Sulphuric acid. |
| 1st day | 226.41 | 11760.57 | 5115.07 | 8.10 | 43.42 | 31.43 | 664.20 | 15.37 | 139.62 | 55.36 | 41.56 |
| 2d " | 226.40 | 11973.65 | 5286.25 | 8.05 | 43.05 | 32.86 | 658.31 | 15.41 | 142.86 | 55.16 | 42.53 |
| 3d " | 226.35 | 11428.04 | 4963.41 | 8.11 | 44.10 | 30.19 | 666.00 | 14.29 | 148.54 | 56.92 | 40.60 |
| 4th " | 226.44 | 11467.16 | 4895.50 | 8.08 | 45.03 | 33.15 | 673.29 | 14.03 | 142.75 | 54.79 | 43.26 |
| 5th " | 226.43 | 11745.49 | 5004.26 | 8.09 | 43.69 | 30.52 | 682.58 | 13.81 | 146.52 | 53.65 | 40.35 |
| Average. | 226.40 | 11674.98 | 5052.90 | 8.08 | 43.86 | 31.63 | 668.87 | 14.58 | 144.06 | 55.17 | 41.66 |

The above table, therefore, indicates the quantity of carbonic acid, aqueous vapor, fæces, urine and its principal constituents excreted, when the weight of the body was nearly uniform, and when no alcohol was taken into the system. During the continuance of these experiments, my general health was excellent. My pulse averaged eighty-one per minute, and was of moderate strength and fulness. My appetite was good, and digestion was performed with regularity.

Having thus ascertained the state of the system as far as my inquiries advanced, when no alcohol was ingested, and when the food was sufficient to sustain the well-being of the organism, I next proceeded to investigate the action of the substance under consideration when all the circumstances which governed the preceding experiments were observed. On the day succeeding their termination, I commenced the second series by taking four drachms of alcohol at each meal, which course was continued for five days. The alcohol was diluted with an equal quantity of water. The other food, and the mental and physical exercise, sleep, &c., remained undisturbed. The mean temperature of the atmosphere was 72.44°.

The annexed table exhibits the results.

| | Weight of body. | Carbonic acid expired. | Aqueous vapor expired. | Fæces. | URINE. Quantity. | Free acid. | Urea. | Uric acid. | Chlorine. | Phosphoric acid. | Sulphuric acid. |
|---|---|---|---|---|---|---|---|---|---|---|---|
| 1st day | 226.64 | 10527.65 | 4729.52 | 7.11 | 41.90 | 30.17 | 591.10 | 13.21 | 112.15 | 33.29 | 30.87 |
| 2d " | 226.95 | 10474.29 | 4853.27 | 6.91 | 39.71 | 29.29 | 585.17 | 13.18 | 119.10 | 35.40 | 25.84 |
| 3d " | 226.80 | 10256.47 | 4825.33 | 6.79 | 40.24 | 29.78 | 562.20 | 13.98 | 94.70 | 28.47 | 30.19 |
| 4th " | 227.06 | 10175.36 | 4893.68 | 6.76 | 39.88 | 31.65 | 586.52 | 13.24 | 105.38 | 30.17 | 26.24 |
| 5th " | 226.81 | 10289.11 | 4975.19 | 6.75 | 40.45 | 34.26 | 583.41 | 13.12 | 101.04 | 26.18 | 28.18 |
| Average. | 226.85 | 10344.57 | 4855.39 | 6.86 | 40.43 | 31.03 | 581.68 | 13.34 | 106.47 | 30.70 | 28.26 |

Thus, after the use of sixty drachms of alcohol in five days, my weight is seen to have increased from an average of 226.40 pounds to an average of 226.85 pounds, being .45 of a pound difference. The carbonic acid and vapor of water in the expired air had respectively decreased 1324.50 and 196.51 grains, the fæces 1.22 ounces, the urine 3.43 ounces, the urea 87.19 grains, the chlorine 37.59 grains, the phosphoric acid 24.47 grains, and the sulphuric acid 13.40 grains. The free acid and uric acid (especially the former) were so slightly affected as to render it probable that the alcohol had exercised no influence upon them.

The cutaneous transpiration did not appear to be sensibly affected, except upon the third day, when I thought I perceived that it was augmented.

During these experiments, my general health was somewhat disturbed. My pulse was increased to an average of ninety per minute, and was fuller and stronger than previously ; there was headache and increased heat of the skin, and my mental faculties were certainly not so clear as on the days when no alcohol was taken. There was also general lassitude, and indisposition to exertion of any kind. My appetite was variable. Digestion was effected as well as previously. The amount of flatus discharged from the intestines was sensibly diminished.

The metamorphosis of tissue and fat was evidently considerably retarded, as is shown in the decreased amount of urea, &c., excreted by the kidneys, and in the lessened quantity of carbonic acid and aqueous vapor given off in respiration. The diminution in the weight of the fæces was doubtless mainly owing to the increased assimilation of food induced by the alcohol.

As this substance is incapable of being converted into tissue, the increase in the weight of the body was probably owing to the three following causes:

1st. The retardation of the decay of the tissues.

2d. The diminution in the consumption of the fat.

3d. The increase in the assimilative powers of the system by which the food was more completely appropriated and applied to the formation of tissue.

From a due consideration of the foregoing experiments, I am disposed to think that, when the food is sufficient for the requirements of the system, alcohol is injurious by exciting the circulation and tending to produce a plethoric habit of body. In these respects, its influence is no worse than an excessive amount of food of any kind, or the omission of physical exercise when the system is habituated to its use.

It has been repeatedly shown that muscular exertion accelerates the destruction of the tissues, and Böcker has conclusively proven that the action of water is similar. When, therefore, the aliment ingested is sufficient to maintain the strength and weight of the body, alcohol, if indulged in should be counteracted in its effects by one or other of the above compensating influences. The action of chloride of sodium is also antagonistic to that of alcohol, and might be similarly employed. By these means the balance of the organism would be preserved.

It is very evident, however, on a careful review of the preceding investigations, that under many circumstances in which man is unfrequently placed, alcohol might be productive of very beneficial results. The ensuing experiments tend to confirm this observation.

2d. The action of alcohol when the body lost weight from deficiency of food.

I ascertained, that, by reducing the amount of bread daily taken to twelve ounces, and the meat to ten ounces, the loss of weight in the body was sufficiently well marked. I, therefore, after allowing five days to elapse since the last experiments, instituted another series in which I took two ounces less of each of these substances at each meal. The remaining conditions of food, exercise, &c., continued as in the last series. On the evening previous to commencing these observations my weight was 226.73. The mean temperature of the atmosphere was 73.17° The following table shows the results of the experiments in detail.

| | Weight of body. | Carbonic acid expired. | Aqueous vapor expired. | Fæces. | URINE. | | | | | | |
|---|---|---|---|---|---|---|---|---|---|---|---|
| | | | | | Quantity. | Free acid. | Urea. | Uric acid. | Chlorine. | Phosphoric acid. | Sulphuric acid. |
| 1st day | 226.62 | 11125.54 | 4759.82 | 6.02 | 42.80 | 28.55 | 621.50 | 12.84 | 126.26 | 44.30 | 35.33 |
| 2d " | 226.30 | 10862.29 | 4681.59 | 5.98 | 41.27 | 27.49 | 635.22 | 12.72 | 135.37 | 44.10 | 36.57 |
| 3d " | 225.92 | 10555.70 | 4600.18 | 6.05 | 41.55 | 30.45 | 630.43 | 12.74 | 130.29 | 46.08 | 38.82 |
| 4th " | 225.69 | 10641.65 | 4610.25 | 5.96 | 40.10 | 26.17 | 641.34 | 12.55 | 120.45 | 45.51 | 38.68 |
| 5th " | 225.34 | 10686.90 | 4687.28 | 6.00 | 40.76 | 26.35 | 618.18 | 12.95 | 129.13 | 42.24 | 37.48 |
| Average. | 225.97 | 10774.41 | 4667.82 | 6.00 | 41.29 | 27.80 | 629.33 | 12.76 | 128.00 | 44.44 | 37.37 |

During these experiments, my pulse averaged eighty-eight per minute. My general health appeared to be good, except that after exertion I was more exhausted than on the days when full food was taken. My desire for aliment was very much increased, and was never completely appeased by the quantity ingested. The sensible perspiration did not appear to vary from the quantity excreted during the first observations.

I proceeded in the next place to ascertain the effects of alcohol upon my system under circumstances similar to those which existed during the last experiments. With this view I took, on the ensuing day, twelve drachms of alcohol (four drachms at each meal,) and continued it for five days. The mean temperature of the atmosphere was 73.34°. The accompanying table exhibits the results.

| | Weight of body. | Carbonic acid expired. | Aqueous vapor expired. | Fæces. | URINE. | | | | | | |
|---|---|---|---|---|---|---|---|---|---|---|---|
| | | | | | Quantity. | Free acid. | Urea. | Uric acid. | Chlorine. | Phosphoric acid. | Sulphuric acid. |
| 1st day | 225.45 | 10055.72 | 4426.18 | 5.85 | 40.22 | 30.10 | 584.75 | 14.01 | 119.17 | 38.50 | 33.17 |
| 2d " | 225.56 | 9821.91 | 4252.75 | 5.81 | 39.52 | 28.56 | 561.52 | 13.82 | 115.24 | 36.42 | 34.29 |
| 3d " | 225.50 | 10024.60 | 4385.90 | 5.82 | 39.10 | 26.14 | 575.19 | 14.05 | 116.91 | 32.19 | 31.42 |
| 4th " | 225.52 | 9948.25 | 4449.68 | 5.80 | 40.00 | 27.19 | 570.28 | 14.00 | 118.14 | 37.10 | 29.86 |
| 5th " | 225.48 | 9876.18 | 4364.36 | 5.76 | 40.77 | 31.24 | 582.35 | 14.06 | 120.43 | 34.48 | 27.57 |
| Average | 225.50 | 9945.33 | 4375.77 | 5.81 | 39.92 | 28.64 | 574.82 | 13.99 | 117.98 | 35.74 | 31.26 |

During the experiments immediately preceding these, my weight decreased an average of .28 of a pound daily, falling from 226.73 pounds to 225.34. In the present series, under the same conditions, except the use of the alcohol, this decrease has not only been overcome, but, there is an actual average daily increase of .03 of a pound, the weight rising from 225.34 to a mean of 225.50 pounds. The mean weight of the body is less than the mean of the last series, owing to the fact that the average daily gain is not so great as the previous average daily loss.

The carbonic acid expired is seen to have decreased an average of 729.08 grains, the aqueous vapor 312.06 grains, the fæces .19 of an ounce, the quantity of urine 1.37 ounces, the urea 54.51 grains, the chlorine 10.08 grains, the phosphoric acid 8.70 grains, and the sulphuric acid 6.11 grains. The free acid of the urine, and the uric acid, were apparently slightly increased.

The sensible perspiration was not perceptibly effected through the day, but at night, it seemed to be somewhat increased. The general condition of my system was never better. My pulse had fallen to an average of eighty-three per minute, there was no headache, the intellectual faculties were clear, and of normal energy, the quantity of food ingested fully satisfied the appetite, sleep was sound and refreshing, and, in fact, all the functions of the organism were performed with regularity. The absence of any symptoms indicating derangement of the health cannot, I think, be ascribed to immunity by continued use of the alcohol, as ten days had elapsed between the two sets of experiments in which it was taken.

The good effects of this substance in limiting the waste of the body when the supply of food is not sufficient to maintain the vigor of the system, are here very evident, and stand in marked contrast to its influence when an abundance of food was ingested. The strength was not only sustained, but the body gradually, but noticeably gained weight. In short, the alcohol had taken the place of the bread and meat omitted, and, at no apparent disadvantage to the general economy. As a compensating agent for a deficiency of food its power cannot, I think, be questioned.

3d. The effect of alcohol when the body gained weight from excess of food.

For the purpose of ascertaining the action of alcohol under the above condition of the system, I increased the quantity of meat daily eaten, from sixteen to twenty-two ounces, and the bread, from eighteen to twenty-four ounces. By this addition to the amount of aliment, I found my weight underwent a sensible and tolerably regular increase. The remaining food, and mental and physical exertion continued as in the first experiments. Five days were suffered to elapse between this and the last series of investigations. The mean temperature of the atmosphere was 72.06°. The annexed table contains the results of the observations made under the above circumstances.

| | Weight of body. | Carbonic acid expired. | Aqueous vapor expired. | Fæces. | Urine. | | | | | | |
|---|---|---|---|---|---|---|---|---|---|---|---|
| | | | | | Quantity. | Free acid. | Urea. | Uric acid. | Chlorine. | Phosphoric acid. | Sulphuric acid. |
| 1st day | 225.61 | 11872.54 | 4895.32 | 12.10 | 43.30 | 35.63 | 698.70 | 17.25 | 152.18 | 58.29 | 50.67 |
| 2d " | 225.85 | 12251.86 | 5324.48 | 12.98 | 45.61 | 34.71 | 721.62 | 16.82 | 155.21 | 64.06 | 50.18 |
| 3d " | 226.15 | 12329.47 | 5250.79 | 12.74 | 45.23 | 38.23 | 710.43 | 17.86 | 150.59 | 62.10 | 51.50 |
| 4th " | 226.36 | 12178.22 | 5387.20 | 12.76 | 46.18 | 35.45 | 735.84 | 18.05 | 158.25 | 66.75 | 48.15 |
| 5th " | 226.59 | 12165.94 | 5419.68 | 12.65 | 45.56 | 39.28 | 728.37 | 18.10 | 161.03 | 62.38 | 49.05 |
| Average | 226.11 | 11577.61 | 5255.49 | 12.64 | 45.17 | 36.66 | 718.99 | 17.61 | 155.45 | 62.71 | 49.91 |

At 10 o'clock on the night before the commencement of the above experiments my weight was 225.50; a slight diarrhea which occurred in the interval, had probably rendered it somewhat less than it would otherwise have been. On the last day of the series it was 226.59, showing an increase of 1.09 pounds, which, as all the excreted substances had increased in quantity over the amounts of the first series, could have arisen from no other cause than from the excess of food. The sensible perspiration was, also, apparently augmented both by day and night.

Symptoms of derangement of the health were more or less present during the continuance of the observations. The pulse was increased in fulness and frequency, averaging 92 per minute. There was almost constant headache, indisposition to exertion, and increased desire for sleep, which was, however, frequently disturbed by unpleasant dreams. My appetite was not very good, and after eating there was occasional pain. There was an increased discharge of flatus from the intestines.

On the day succeeding the termination of these investigations, I commenced the following by taking, under the conditions of food, &c., of the last experiments, the fixed quantity of four drachms of alcohol at each meal, which, as previously, was continued for five days. The ensuing table exhibits the results. The mean temperature of the atmosphere was 73.60°.

| | Weight of body. | Carbonic acid expired. | Aqueous vapor expired. | Fæces. | URINE. | | | | | | |
|---|---|---|---|---|---|---|---|---|---|---|---|
| | | | | | Quantity. | Free acid. | Urea. | Uric acid. | Chlorine. | Phosphoric acid. | Sulphuric acid. |
| 1st day | 226.82 | 12015.37 | 5384.47 | 10.40 | 40.91 | 38.11 | 627.58 | 18.20 | 128.35 | 49.82 | 38.17 |
| 2d " | 227.17 | 11523.19 | 5090.26 | 10.22 | 40.50 | 36.34 | 639.60 | 18.31 | 121.42 | 51.27 | 39.72 |
| 3d " | 227.48 | 11452.71 | 4829.64 | 10.38 | 41.37 | 34.13 | 629.41 | 18.11 | 126.15 | 46.14 | 40.55 |
| 4th " | 227.80 | 11514.28 | 4831.70 | 10.18 | 40.62 | 39.24 | 610.17 | 18.01 | 135.10 | 46.98 | 40.52 |
| 5th " | 228.15 | 11382.50 | 4810.35 | 10.35 | 41.73 | 35.46 | 621.86 | 18.15 | 131.68 | 47.51 | 36.23 |
| Average | 227.48 | 11577.61 | 4989.28 | 10.30 | 41.02 | 36.65 | 625.72 | 18.15 | 128.54 | 48.34 | 39.04 |

During the series of experiments immediately preceding the present, the average daily increase of weight was .22 of a pound. By the above table, it is seen that, by the action of the amount of alcohol ingested, the average increase was raised to .31 of a pound per day. The average amount of carbonic acid excreted, compared with the mean of the last series, was reduced 581.99 grains, the aqueous vapor 266.21 grains, the fæces 2.34 ounces, the urine 4.15 ounces, the urea 93.27 grains, the chlorine 26.92 grains, the phosphoric acid 8.29 grains, and the sulphuric acid 14.87 grains. The free acid and uric acid were but slightly affected. The perspiration was sensibly diminished.

Whilst these experiments were progressing, the healthy action of my system was very much disordered. Headache was constant, sleep was disturbed, the skin was hot, pulse full and bounding, averaging 98 per minute, and there was on two occasions after eating slight palpitation of the heart. My appetite was capricious. Sometimes disgust was created by the mere sight of food, at other times I ate with a good deal of relish. I think I should have been made seriously ill if I had continued the investigations longer. Upon a return, however, to my ordinary food, all unpleasant symptoms gradually disappeared. This fortunate termination was probably promoted by a diarrhea of considerable violence, which commenced on the second day after the conclusion of the experiments, and continued forty-eight hours.

The inquiries into the actions of alcohol upon the human economy were now terminated. Upon consideration of the foregoing experiments collectively, I arrive at the conclusion, *that alcohol increases the weight of the body by retarding the metamorphosis of the old tissues, promoting the formation of new, and limiting the consumption of the fat.* Viewed in detail, it is seen that, under the use of alcohol, the following effects constantly ensued:

1st. The carbonic acid and aqueous vapor given off in respiration were lessened in quantity.

2d. The amount of fæces was diminished.

3d. The quantity of urine was reduced.

4th. The urea, chlorine, and phosphoric and sulphuric acids were diminished in amount.

These effects, occurring when the amount of food was below the quantity required to maintain the weight of the body under the mental and physical exercise taken, were productive of no deleterious results to the system. On the contrary, when the food was sufficient to balance the waste from the excretions, and still more so when an excess of aliment over the demands of the organism was ingested, the healthy working of the system was disturbed, and actual disease almost induced.

The use of alcohol, even in moderation, cannot, therefore, be either exclusively approved or condemned. The laboring man, who can hardly procure bread and meat enough to preserve the balance between the formation and decay of his tissues, finds here an agent which, within the limits of health, enables him to dispense with a certain quantity of food, and yet keeps up the strength and weight of his body. On the other hand, he who uses alcohol when his food is more than sufficient to supply the waste of tissue, and, at the same time, does not increase the amount of his physical exercise, or drink an additional quantity of water (by which the decay of tissue would be accelerated,) retards the metamorphosis whilst an increased amount of nutriment is being assimilated, and thus adds to the plethoric condition of the system, which excessive food so generally induces.

The foregoing experiments confirm those of Böcker so far as the diminution of the carbonic acid expired, and the reduction of the solids and water of the urine are concerned. This physiologist, however, found that under the use of alcohol the fæces excreted and the water exhaled from the lungs were unaffected. The present investigations, on the contrary, indicate that both the fecal excretion and the water expired were materially diminished. These discrepancies are probably due to the difference in the quantities of alcohol imbibed, the preceding experiments being performed with a much larger amount of this substance than were Böcker's.

The perspiration not having been measured by direct experiment, I have not laid much stress upon the apparent results obtained. The temperature of the atmosphere was, however, unusually uniform during the continuance of the observations, and any alteration in the quantity of this excretion was doubtless owing to the influence of the alcohol. Yet the liability to form an erroneous opinion, when judging only from the sensations, leaves the action of alcohol upon the cutaneous transpiration still to be definitely determined.

It has been assumed by several late writers that the primary action of alcohol is the retention in the blood of the products of metamorphosis. I am inclined to think this opinion erroneous, and that alcohol, instead of preventing the elimination of the decayed tissues, acts by preventing, in a great measure, their primary destruction. No one will dispute the point that, if the first of these views is correct, alcohol must be uniformly deleterious, and that it must manifest such unmistakeable symptoms as could not possibly lead to a misconstruction of its mode of action. If this had been its influence on my own system, what an immense accumulation of carbonized and nitrogenized substances would have been retained in the blood, and what a different set of symptoms would have been experienced! Besides, these symptoms would have been also present during the experiments conducted with alcohol when an insufficient quantity of food was taken; and yet on these days they were entirely absent, and my system was never in better order. Indeed, it may possibly be a question of doubt in the minds of some whether the unpleasant symptoms which were observed were not due as much to excessive food as to the alcohol.

The most strenuous supporter of the theory that alcohol causes the retention of the decomposed tissues in the blood is Dr. Carpenter, and it is with great diffidence that I find myself constrained to differ with so eminent a physiologist. Dr. Carpenter, also, whilst admitting (*Essay on Alcohol*) that there are occasions when it is of importance that an increased amount of mental or physical exertion should be made, and that under such circumstances alcohol may be temporarily beneficial, ascribes its influence in producing additional nervous force to the fact that it occasions more rapid metamorphosis of the nervous tissues. The experiments detailed in the present paper invariably show a diminished excretion of the products of nervous decay after the exhibition of alcohol, and consequently such cannot be its action.

I do not wish to be understood as at all contending for the propriety of habitual indulgence in alcohol. My experiments show that there are circumstances in which its use is injurious. I believe, however, that these circumstances can be so modified that alcohol may be moderately indulged in without the production of deleterious effects. Full food, insufficient exercise, and alcohol conjoined, will as certainly produce disease if the action of this latter agent is the retardation of tissue-metamorphosis, as though it prevented the elimination from the blood of substances injurious to the organism. On the one hand, however, the affection would be of a sthenic, and on the other of an asthenic character. Whilst, therefore, fully admitting that the use of alcohol requires prudence and discretion, I am not prepared to concede that it is essentially poisonous, or even that there are not conditions of the system in which its employment is not eminently to be commended.

*Tobacco.*—The experiments with this substance, though not so full as those with alcohol, were conducted upon the same general principles. They embraced the consideration of its effects under the following conditions:

1st. When the food was sufficient to maintain the healthy balance of the system.

2d. When a deficiency of aliment was ingested.

I had previously instituted some experiments, which, though incomplete, were sufficient to indicate the general action of tobacco upon the organism. They were conformatory of the present so far as they extended, which was principally to the relations of the substance under consideration to the urine and its constituents.

As in the experiments with alcohol, I fixed upon a definite and invariable amount of sleep and mental and physical exertion. This was precisely as has been previously stated in detail. The experiments related to the same determinations, and all the analyses were performed in exactly the same manner, and at the same periods of the day as formerly.

After the expiration of twelve days since the investigations into the action of alcohol, and when my system was again in a perfectly normal condition, I commenced the experiments with tobacco. I am not in the habit of using this substance in any form, but had, previous to my observations, smoked an occasional cigar without any perceptible effect resulting, other than slight nervous excitement. I have never in my life chewed tobacco or used snuff.

1st. The effects of tobacco when a sufficiency of food was taken to keep up the weight and vigor of the body.

I lived exactly in the corresponding series of experiments with alcohol, except that I found it necessary, from, as I suppose, the greater heat of the atmosphere, and consequently the induction of a larger amount of cutaneous transpiration, to increase the quantity of water from forty-eight ounces daily to fifty-two ounces—thirteen at each meal, and thirteen immediately before going to bed. The observations under this mode of living were continued, as before, for five days. The mean temperature of the atmosphere for the period was 80.12°. The following table exhibits the results :

| | Weight of body. | Carbonic acid expired. | Aqueous vapor expired. | Fæces. | URINE. | | | | | | |
|---|---|---|---|---|---|---|---|---|---|---|---|
| | | | | | Quantity. | Free acid. | Urea. | Uric acid. | Chlorine. | Phosphoric acid. | Sulphuric acid. |
| 1st day | 225.84 | 11845.29 | 4827.50 | 8.12 | 40.54 | 29.43 | 643.18 | 13.10 | 151.62 | 57.42 | 39.52 |
| 2d " | 225.78 | 11582.73 | 4855.91 | 8.10 | 41.66 | 27.82 | 662.27 | 12.78 | 155.16 | 54.38 | 36.18 |
| 3d " | 225.76 | 11628.65 | 4986.70 | 8.11 | 42.13 | 30.51 | 650.30 | 12.64 | 144.25 | 52.29 | 35.27 |
| 4th " | 225.80 | 11439.26 | 4758.37 | 8.07 | 42.76 | 26.17 | 665.14 | 12.82 | 142.51 | 56.77 | 35.40 |
| 5th " | 225.76 | 11586.40 | 4994.85 | 8.09 | 41.35 | 25.39 | 667.58 | 12.80 | 150.52 | 60.06 | 38.22 |
| Average | 225.79 | 11616.46 | 4884.66 | 8.10 | 41.69 | 27.86 | 657.69 | 12.83 | 148.81 | 56.18 | 36.92 |

The heat of the atmosphere during the above experiments was 7.06 degrees greater than during the first set of experiments in the alcohol series. My pulse averaged 85 per minute. My health, notwithstanding the extreme heat of the weather, was excellent. My appetite was good, and my food was well digested.

Under the same conditions as the experiments just concluded, I proceeded in the next place to ascertain the direct effects of tobacco. With this object, I smoked one hundred and fifty grains of tobacco (nearly two cigars) after each meal, being four hundred and fifty grains per day. During these experiments, the mean temperature of the atmosphere was 78.11°. The annexed table exhibits the results.

| | Weight of body. | Carbonic acid. expired. | Aqueous vapor expired. | Fæces. | URINE. | | | | | | |
|---|---|---|---|---|---|---|---|---|---|---|---|
| | | | | | Quantity. | Free acid. | Urea. | Uric acid. | Chlorine. | Phosphoric acid. | Sulphuric acid. |
| 1st day | 225.81 | 11726.58 | 4658.22 | 8.10 | 40.21 | 30.84 | 628.41 | 18.29 | 135.43 | 88.60 | 40.59 |
| 2d " | 225.87 | 11562.97 | 4473.18 | 8.11 | 39.63 | 32.26 | 610.93 | 18.80 | 118.15 | 84.10 | 43.17 |
| 3d " | 225.86 | 11839.65 | 4485.41 | 8.09 | 39.80 | 35.18 | 614.11 | 19.03 | 127.84 | 75.33 | 38.65 |
| 4th " | 225.90 | 11710.80 | 4627.64 | 8.06 | 39.45 | 31.59 | 604.50 | 19.01 | 117.25 | 81.52 | 40.10 |
| 5th " | 225.85 | 11482.51 | 4681.57 | 8.10 | 40.02 | 34.57 | 618.68 | 18.45 | 130.21 | 70.49 | 44.15 |
| Average | 225.86 | 11664.50 | 4585.20 | 8.09 | 39.82 | 32.89 | 615.32 | 18.71 | 125.77 | 80.01 | 41.33 |

Under the use of tobacco, my weight had increased an average of .07 of a pound, the carbonic acid 88.04 grains, the free acid of the urine 4.93 grains, the uric acid 5.88 grains, the phosphoric acid 23.83 grains, and the sulphuric acid 4.41 grains. On the contrary, the quantity of aqueous vapor had decreased 299.46 grains, the fæces .01 of an ounce, the urine 1.87 ounces, the urea 42.37 grains, and the chlorine 23.04 grains.

The general effects of the tobacco upon my system were exceedingly well marked. There was great nervous excitement accompanied by irregular action of the muscles, more particularly of the eyelids, mouth, and upper extremities, which lasted for about two hours after each occasion of using this substance. The mind, however, was clear, and there was no headache. These sensations were succeeded by a pleasant feeling of ease and contentment, which also lasted about two hours. During the first part of the night, there was wakefulness, but this was always followed by a sound sleep, which continued till the hour for rising. The pulse was increased to an average of 92 per minute. My appetite was as good as usual. The perspiration was apparently slightly diminished.

After allowing five days to elapse, as in former experiments, in order that the system might have time to regain its natural condition, I commenced the observations under the second head, viz. the *effects of tobacco upon the organism, when an insufficiency of food was taken.* I reduced (as in the corresponding experiments with alcohol) the quantity of bread daily ingested to twelve ounces, and the meat to ten ounces. In all other respects, the conditions of the last experiments remained unaltered. During these investigations the mean temperature of the atmosphere was 80.92°. The results are contained in the following table:—

| | Weight of body. | Carbonic acid expired. | Aqueous vapor expired. | Fæces. | URINE. | | | | | | |
|---|---|---|---|---|---|---|---|---|---|---|---|
| | | | | | Quantity. | Free acid. | Urea. | Uric acid. | Chlorine. | Phosphoric acid. | Sulphuric acid. |
| 1st day | 225.58 | 10672.86 | 4537.69 | 6.02 | 38.74 | 22.47 | 623.50 | 11.58 | 128.31 | 45.78 | 34.54 |
| 2d " | 225.20 | 10384.61 | 4483.22 | 6.06 | 38.20 | 24.18 | 615.11 | 11.23 | 131.58 | 43.29 | 33.09 |
| 3d " | 224.79 | 10350.92 | 4394.48 | 6.04 | 39.04 | 25.72 | 604.25 | 10.01 | 125.44 | 44.64 | 32.22 |
| 4th " | 224.33 | 10526.45 | 4456.73 | 6.03 | 39.57 | 26.19 | 601.18 | 9.82 | 130.17 | 42.18 | 30.15 |
| 5th " | 223.97 | 10347.81 | 4375.16 | 6.05 | 38.73 | 24.65 | 608.46 | 10.04 | 132.26 | 45.25 | 28.31 |
| Average. | 224.77 | 10456.53 | 4449.45 | 6.04 | 38.85 | 24.64 | 610.50 | 10.53 | 129.55 | 44.23 | 31.66 |

The general effects observed, were of a similar character to those noticed during the experimemts performed under like conditions in the alcohol series. The extreme heat of the weather, however, rendered the amount of sensible perspiration much larger. The pulse was 86 per minute. My appetite was always good; but as I always left the table with a feeling of hunger, I felt myself gradually becoming weaker day by day. On the night previous to the commencement of these experiments, my weight was 225.81. On the last day of the series it was 223.97 I had, therefore, lost 1.84 pounds, or an average daily of nearly .37 of a pound.

Under the condition of the system thus produced, I began, on the day following the conclusion of the experiments just detailed, and under circumstances every way identical, the concluding series relative to the effects of tobacco. I smoked, as previously, one hundred and fifty grains of cigars after each meal. The average temperature of the atmosphere was 74.09°.

The special results are exhibited in the annexed table.

| | Weight of body. | Carbonic acid expired. | Aqueous vapor expired. | Fæces. | URINE. | | | | | | |
|---|---|---|---|---|---|---|---|---|---|---|---|
| | | | | | Quantity. | Free acid. | Urea. | Uric acid. | Chlorine. | Phosphoric actk. | Sulphuric acid. |
| 1st day | 223.80 | 10568.37 | 4382.28 | 4.50 | 37.85 | 26.81 | 569.70 | 14.91 | 118.36 | 72.86 | 40.21 |
| 2d " | 223.65 | 10495.13 | 4417.30 | 4.48 | 37.29 | 25.14 | 541.28 | 15.11 | 111.53 | 75.71 | 38.02 |
| 3d " | 223.55 | 10265.80 | 4293.74 | 4.49 | 37.15 | 28.16 | 536.12 | 15.29 | 115.83 | 78.60 | 39.00 |
| 4th " | 223.56 | 10483.69 | 4150.83 | 4.53 | 37.48 | 28.73 | 552.10 | 14.80 | 112.40 | 74.22 | 42.23 |
| 5th " | 223.54 | 10478.36 | 4203.41 | 4.62 | 36.92 | 29.54 | 540.61 | 15.17 | 114.66 | 70.91 | 40.58 |
| Average | 223.62 | 10458.27 | 4289.51 | 4.52 | 37.34 | 27.67 | 547.96 | 15.05 | 114.55 | 74.46 | 40.01 |

From the above table, it is seen that the loss of weight in the body, induced by the deficient supply of food, was lessened from the first, and entirely overcome on the fourth day—the average daily loss being less than .09 of a pound, against .37 of a pound, under the same conditions, except the use of tobacco. The excretion of carbonic acid from the lungs was not, in the average, perceptibly affected. The amount of aqueous vapor exhaled was reduced 154.94 grains, the fæces 1.92 ounces, the quantity of urine 1.51 ounces, the urea 62.54 grains, and the chlorine 15 grains. The free acid of the urine was increased 3.03 grains, the uric acid 4.52 grains, the phosphoric acid 30.23 grains, and the sulphuric acid 8.35 grains.

The general effects upon the system were almost identical with those previously described as resulting from the former use of tobacco. There was the same nervous excitement, trembling, and wakefulness, but in a somewhat less degree. The pulse was an average of 90 per minute. The desire for food was not nearly so great as in the last experiments, neither was there so great a degree of debility. The cutaneous transpiration, whether from the diminished temperature of the atmosphere, or as an effect of the tobacco used, was very sensibly lessened in quantity.

From these experiments the following conclusions are deducible :—

1st. That tobacco does not materially affect the excretion of carbonic acid through the lungs.

2d. That it lessens the amount of aqueous vapor given off in respiration.

3d. That it diminishes the amount of the fæces.

4th. That it lessens the quantity of urine, and the amount of its urea and chlorine.

5th. That it increases the amount of free acid, uric acid, and phosphoric and sulphuric acids, eliminated through the kidneys.

These results differ in several essential points from those obtained with alcohol. The fact that the amount of carbonic acid given off in respiration was not diminished, would indicate that the consumption of the fat of the body is not lessened by the use of tobacco. The metamorphosis of the nitrogenous tissues, judging from the diminution in the quantity of urea and chlorine observed, would appear to be retarded, and yet the amount, both of the phosphoric and sulphuric acids excreted, especially the former, was very considerably augmented. As both phosphorus and sulphur enter into the composition of all the proteinaceous tissues, it is difficult to reconcile this apparent inconsistency in the results, unless by assuming (what there is great reason to believe) that the oxydation of the phosphorus, and sulphur of the brain, and nervous tissue, was so great in amount as to cause an increase in the elimination of phosphoric and sulphuric acids, even though the metamorphosis of the other nitrogenous tissues was lessened.

The effect produced by tobacco upon the excretion of the free acid, and uric acid of the urine, was also different from that caused by alcohol. Though both alcohol and tobacco diminish the quantity of urea, the latter only of these substances would appear to excercise any very material influence upon the amount of uric acid eliminated. If there are any definite and constant relations existing between these two constituents of the urine, they would appear to be farther from determination than ever.

Tobacco, when the food is sufficient to preserve the weight of the body, increases that weight, and when the food is not sufficient, and the body in consequence loses weight, tobacco restrains that loss. Unlike alcohol, this influence is unattended with any unpleasant effects upon the circulatory system, though its action on the brain and nerves is certainly not such as always to be desired. When used in greater moderation than in these experiments, this influence would, doubtless, be greatly lessened.

I refrain from entering into the discussion of the other physiological points connected with the foregoing experiments. A simple examination of the tables will show that these are many and of great interest, and that it is not only as exhibiting the actions of alcohol and tobacco upon the system that the investigations detailed in this paper are valuable; neither have I the time to discuss farther the immediate subjects of inquiry.

To that earnest band of physiologists who are constantly investigating the operations of nature, and who rely more upon actual observations than upon abstract theories, I submit these experiments. Though the deductions I have drawn from them may not stand before the progress of physiological research, the materials collected will, I am confident, never entirely lose their value.—*Am. Jour. of Med. Sci.*

